# Supplementary material for: Fungal Hyphae on the Assimilation Branches Are Beneficial for Haloxylon ammodendron to Absorb Atmospheric Water Vapor: Adapting to an Extreme Drought Environment
Source: Plants (Basel). 2024 Apr 29;13(9):1233. doi: 10.3390/plants13091233 (PMC11085276; doi:10.3390/plants13091233)
Supplement: Supplementary file 1 [file plants-13-01233-s001.zip › plants-2884526-supplementary.pdf]

Table S1 Statistical table of atmospheric relative humidity above 50% during the growing season from May to September 2019 in Ejina Banner

RH (%)	Total time （min）	Minutes/Day (min/d)	Hours/Day (h/d)	Percent (%)
50≤RH<60	10070.00	65.82	1.10	36.66
60≤RH<70	6900.00	45.10	0.75	25.12
70≤RH<80	4370.00	28.56	0.48	15.91
80≤RH<90	5180.00	33.86	0.56	18.86
90≤RH≦100	950.00	6.21	0.10	3.46
sum	27470.00	179.54	2.99	100.00
